# Use of physical restraint in hospital patients: A descriptive study in a tertiary hospital in South Africa

**DOI:** 10.4102/curationis.v39i1.1605

**Published:** 2016-11-10

**Authors:** Sebastiana Z. Kalula, Sabela G. Petros

**Affiliations:** 1 Division of Geriatric Medicine, The Albertina and Walter Sisulu Institute of Ageing in Africa, Department of Medicine, University of Cape Town, South Africa; 2 Department of Medicine, The Albertina and Walter Sisulu Institute of Ageing in Africa, University of Cape Town, South Africa; 3 Provincial Department of Health, Western Cape Province, South Africa

## Abstract

**Background:**

The use of physical restraint in patient management is a common and emotive issue, and has legal and ethical dimensions.

**Objective:**

To document the prevalence of physical restraint use, patient characteristics associated with physical restraint use, and nurses’ and doctors’ knowledge and perceptions towards the practice.

**Methods:**

A cross-sectional study of 572 patients, of whom 132 were physically restrained, was conducted in acute wards of a tertiary hospital. Data were collected on the 132 physically restrained patients. Fifty-nine doctors and 159 nurses completed a specially constructed questionnaire. Descriptive statistics were derived and expressed as numbers and percentages.

**Results:**

Prevalence of restraint use was 23% (132/572). The distribution in acute wards was: medical 54.5%; surgical 44.7%; maternity 0.8%; psychiatry none. Mean age (SD) of the restrained patients was 49 years (20.5); 53.8% were male. The commonest types of restraints used were bed rails 93% and wrist belts 12%. Restraints were used largely to protect medical devices and as protection from harm. Less than 15% of the nurses reported having received training and 36% of the doctors reported having received some guidance on the use of restraints. Only a minority of nurses and doctors knew of a hospital policy on restraint use. Documentation on the prescription and indication for the use of restraint was poor.

**Conclusion:**

Prevalence of restraint use is high and poorly coordinated. A policy on the use of restraint and comprehensive guidelines should be developed to guide health care practitioners in the management of patients where restraint cannot be avoided.

## Introduction

The use of physical restraint in hospital patients has been extensively investigated in so-called developed countries (Cotter 2005; Evans & Strumpf 1989; Heinze, Dassen & Grittner 2012; Krüger *et al*. 2013; Martin & Mathisen 2005), but less commonly in the developing world. Arguments are proffered to both support (Demir 2007; Heinze *et al*. 2012; Martin & Mathisen 2005; Minnick *et al*. 2007), and question, discourage and reject (Agens 2010; Watson 2001) the practice. In the former case it is argued that the practice is purely to ensure patient safety as well as the safety of other patients and health care practitioners (Demir 2007; Heinze *et al*. 2012; Martin & Mathisen 2005; Minnick *et al*. 2007). In the latter case arguments pertain to the practice constituting a violation of patients’ human rights (Sokol 2010).

Restraint of patients in hospitals may be chemical, physical and/or psychological. Physical restraint refers to devices and practices that restrict a patient’s movement (Agens 2010; Benbenbishty, Adam & Endacott 2010; Lane & Harrington 2011). The practice is justified by hospital staff as being in the patient’s interest, as he or she may be at risk of harming him or herself, but equally pose a danger to other patients and health care practitioners (Demir 2007; Heinze *et al*. 2012; Martin & Mathisen 2005; Minnick *et al*. 2007). Other arguments pertain to a risk that the patient may remove a medical device – and litigation that may ensue should the patient sustain injuries or cause injury to others (Griffith 2014; Mohr 2010).

Arguments commonly put forth by hospital personnel for the use of physical restraint in older patients pertain to the prevention of falls, the control of agitated patients, the prevention of their wandering and the protection of medical devices (Chuang & Huang 2007; Fradkin, Kidron & Hendel 1999; Goethals *et al*. 2012; Suen *et al*. 2006). Hospital carers’ attitudes towards the practice may also contribute to its continued use, based on a contention, for example, that the use of physical restraint cannot be eliminated without additional staffing and resources (McCabe *et al*. 2011; Mion 2008; Werner & Mendelson 2001).

The use of restraint in hospital settings is thus an emotive issue (Gastmans & Milisen 2006; Möhler & Meyer 2014; Stubbs *et al*. 2009; Watson 2001) inasmuch as physical, psychological, legal and ethical dimensions are involved (Agens 2010; Gallinagh *et al*. 2001; Watson 2001). Hence, the practice is receiving increasing attention from researchers, health practitioners and concerned citizens (Griffith 2014).

Numerous situations indeed exist in which a patient may be a danger to, and injure, him or herself, and/or others, if not physically restrained. Changed mental status may render a patient unable to comprehend the purpose of treatments necessary for his or her safe care (Irish Nurses Organisation 2003). The self-removal or disruption of devices such as nasogastric tubes, intravenous lines or oxygen masks used for patient management may have disastrous consequences. Failure of the medical team to protect the devices from consequences of patients’ delirious behaviour would be regarded as negligence.

Conversely, a potential for physical restraints to cause harm to patients and others has been reported in a number of studies conducted in developed countries (Evans & Strumpf 1989; Martin & Mathisen 2005). Choices between use and non-use of restraint, it has been argued, should be based on practical considerations, such as how a patient’s safety (and the safety of others) may be balanced with a patient’s swift recovery (Griffith 2014; Möhler & Meyer 2014). Agitated and/or confused patients may indeed have to endure physical and/or chemical restraint treatment to allow the administration of appropriate therapy for fast recovery – and ultimately freedom from restraint (Evans & FitzGerald 2002). Hence, where restraint is used, the risk of a patient’s untoward interference with treatment should outweigh the physical, psychological and ethical risks of its use (Irish Nurses Organisation 2003). Nonetheless, specific forms of restraint can themselves pose a threat for patients through complications that may result. Mechanical forms of restraint, for example, may subject a patient to the risk of physical harm (Choi & Song 2003; Demir 2007; Mion 2008).

The use of physical restraint in older patients has been associated with poor outcomes such as functional and psychological decline (Agens 2010; Bower & McCullough 2000; Strout 2010). Hence, the use of restraint in hospital patients is vexatious. Indeed, hospitals, patients and/or their carers may incur financial costs for the management of injuries and pressure sores resulting from the use of physical restraint, as well as through possible litigation brought against the hospital by family members.

Finally, an area of dispute and concern increasingly forged by human rights activists, in particular, is that of the use of physical and other restraint constituting a violation of patients’ rights (Sokol 2010). More importantly, the continued application of restraint on hospital patients is at odds with a prevailing ethos, in South Africa in this case, which emphasises individual rights and is promoted in the Patients’ Rights Charter. The Patients’ Rights Charter emphasises that health care providers should display a positive disposition that demonstrates courtesy, human dignity, patience, empathy and tolerance towards patients (Health Professions Council of South Africa 2008). Specifically, the charter prescribes that treatments which include the use of a restraint device should be applied in a way that upholds the patient’s dignity. International guidelines on the use of restraint prescribe moreover that both patients and carers must be given appropriate information on the need for restraining therapy (Irish Nurses Organisation 2003).

### Problem statement

Physical and/or chemical restraints are used commonly in acute care settings in public hospitals of Cape Town. It is evident that the decision to use restraints needs careful consideration from those providing the care. As presented in the literature review, extensive research pertaining to the use of restraints as a form of patient management emanates from high income countries. Although restraints are used locally, to our knowledge, no empirical evidence exists in South Africa on the practice, its efficacy and risks involved in the use of physical restraint, or staff perspectives towards the practice. A study was consequently undertaken to document the prevalence of restraint use, and nurses’ and doctors’ knowledge and perceptions towards the practice in acute care wards. It was envisaged that the study would indicate levels of support for its use or non-use within therapeutic management.

There were three main research questions in this study.

- What is the prevalence and type of restraint used in patient management in acute care wards?
- What are the perceptions and knowledge of nurses and doctors towards the use of restraint?
- Is there a hospital policy to guide health care practitioners on the use of restraints?

### Research objectives

- Determine the prevalence of restraint use on acute care wards and complications known to be associated with their use
- Determine the knowledge on restraint use in acute care among nurses and doctors
- Explore and describe nurses’ and doctors’ perceptions on restraint use
- Determine the existence of the hospital’s policy and/or guidelines that direct restraint use
- Restraint was defined as application of a device, material or equipment near to or attached to a patient to deliberately prevent the patient’s free body movement to a position of his or her choice, or to prevent the patient’s access to his or her body (Evans *et al*. 2002; Meyer *et al*. 2009; Köpke *et al*. 2012).

## Methods

### Design

A cross-sectional study was conducted in a tertiary hospital in Cape Town, South Africa. The hospital has 975 beds and 57 884 inpatient admissions annually (Groote Schuur Hospital Fast Facts 2015). The hospital has approximately 242 registrars, 82 interns, 717 registered nurses (RNs) and 330 enrolled nurses (ENs) of whom two-thirds work day shift and one-third night shift. The researcher explored knowledge and perceptions of RNs, ENs, and doctors. The doctors were registrars under specialist training and interns working towards their registration as medical practitioners. Specific wards for in-patient management selected for the study were the medical, surgical, obstetrics and gynaecology, and psychiatry wards that managed acute patients. Data were collected in wards with patients aged 13 years and older (13 year olds are admitted to adult wards) who were found to be physically restrained on days a researcher visited the ward. High care and intensive care wards were excluded because of the differences in patient needs and management from those of the general acute wards. A unique hospital folder number was used to identify each restrained patient, which avoided duplication of data; each patient was documented only once. The total number of patients on the ward on the day of the visit was recorded and information was extracted from the records of restrained patients. Because of varying levels of cognitive function, patients were not interviewed.

Data were collected from a convenience sample of 150 professionally trained nurses working in these wards during the time of the researcher’s visits. A quota sample of 59 medical registrars and interns was drawn from doctors present on the wards at the time the researcher visits. The medical practitioners were selected on grounds of their involvement in daily management decisions of patients. All doctors were registered with the South African Health Professions Council.

All participating nurses were qualified (registered or enrolled) and registered with the South African Nursing Council; all worked full time or part time on the wards. The nurses had been in clinical practice for more than 24 months and had worked in a hospital setting for a minimum of 6 months. Sampled nurses’ and medical practitioners’ participation in the study was voluntary.

### Data collection

The survey was conducted using a pre-constructed questionnaire with both fixed response and open-ended items. A semi-structured questionnaire was developed based on published literature and the Perceptions of Restraint Use Questionnaire (PRUQ) (Capezuti 2004; Evans & Cotter 2008; Gallinagh *et al*. 2001; Miles & Irvin 1992; Strumpf & Evans 1988). The questionnaire comprised demographic variables, dichotomous (yes or no) items, rank order items (most preferred to least preferred) and a Likert type scale (do not approve; approve very little; approve somewhat; approve very much). Different questionnaires were used for doctors and nurses. Modifications to the questionnaire were in the demographic variables section but sections on perceptions on the use of restraint and rights of the patient were the same. The researcher handed the questionnaires to the participants individually in a ward side room and waited to collect the questionnaires upon completion. Information on patient profiles and diagnoses were obtained from medical and nursing notes by a medical practitioner who was part of the research team. The types of restraint used, and indications for their use, as well as complications that arose known to be associated with the use of particular restraints were recorded. The participating wards were aware of the ongoing study but the days and times that wards were visited for data collection were unannounced to prevent modification of patient management. Intermittently, cross-sectional data were collected over the period January to August 2010 in order to include different cadres of nursing staff and doctors. Wards under a specific specialty were visited not more than once in 2 weeks to allow for patient turnover.

### Data analysis

Quantitative data were captured using the Access 2003 statistical package. Statistical package for social sciences (IBM SPSS Statistics for Windows, Version 19) was used to analyse the data. Non-numerical data were converted into numerical codes and analysed quantitatively. Frequency and descriptive statistics were derived and expressed as numbers and percentages.

## Ethical considerations

Permission to conduct the study was obtained from the Human Ethics Committee of the Faculty of Health Sciences at the University of Cape Town (Research Ethics Committee Reference: 122/2009). Written permission to conduct the study was also obtained from the Chief Operations Officer (representing the hospital and four superintendents responsible for the wards participating in the study) and from nursing managers of the hospital and the wards. Written informed consent was obtained from the nurses and doctors prior to participation in the study. The researcher explained the purpose and objectives of the study to potential participants. The informed consent form was given to the participant and the researcher returned approximately 1 hour later to interview those willing to participate in the study. Participants were assured of anonymity and confidentiality of information they divulged. Consent was not obtained from patients; a medical practitioner extracted information from restrained patients’ medical records using a pre-designed questionnaire. All data obtained were labelled with a code number for identification and not a participant’s name.

## Results

### Patients

There were 29 visits to the wards over the period of 8 months (35 weeks). In total, 572 patients were visited and 132 (23%) were found to have some form of restraint. Of the 132 restrained patients, slightly over half were male (Table 1). Mean age (SD) was 49 (20.5) years (range 13–94 years). The distribution was: medical wards 54.5%; surgical wards 44.7%; maternity ward 0.8%; none in the psychiatry ward.

**TABLE 1:** Characteristics of restrained patients (*n* = 132) and type of restraint.

Characteristics	*N* (%)
Age in years, Mean (s.d.)	49 (20.5)
Gender	
Male	71 (53.8)
Female	61 (46.2)
**Age**	
< 45 years	56 (42.4)
45–59 years	34 (25.8)
≥ 60 years	42 (31.8)
Main diagnosis	
Central nervous system disorder	53 (40.0)
Brain trauma	22 (42.0)
Stroke	20 (38.0)
Meningitis or encephalitis	8 (15.0)
Cord and peripheral CNS	3 (5.7)
Cardiac disease	17 (12.9)
Gait disorder	10 (7.6)
Respiratory infection	9 (6.8)
Renal failure	6 (4.5)
Other infection	9 (6.8)
Other trauma	9 (6.8)
Diabetes mellitus	8 (6.1)
Type of restraint (multiple restraints use; percentages exceed 100%)	
Bed rails	123 (93.2)
Wrist straps	16 (12.1)
Bedding	6 (4.5)
Other	10 (7.6)
Indication for restraint	
Confused	32 (24.2)
Agitation	32 (24.2)
Violent or disruptive	11 (0.8)
Other	11 (0.8)
Not stated	66 (50)

*Source:* Authors’ own work

s.d., standard deviation; CNS, central nervous system. Unless otherwise stated, data represent number (*N*) and percentage (%).

Forty-two (31.8%) restrained patients were age ≥ 60 years and 42% of the total sample were younger than 45 years. The main diagnoses of the restrained patients were: central nervous system conditions (40%), subdivided into brain trauma 41.5% (22), stroke 37.7% (20), meningitis or encephalitis 15.1% (8) and cord and peripheral nervous system disorder 5.7% (3) (Table 1). Reasons for the use of restraint in the patients were: confusion (24%), agitation and disruptive or violent behaviour (24%) and post general anaesthesia (13%). No reason was stated for 50% (66/132) of patients. The commonest type of restraint was bed rails (123 (93%)) followed by wrist straps (belts) (16 (12.1%)) (Table 1). Additional chemical restraint was used in 12% of patients: benzodiazepines (34.5%), morphine (38.6%) – largely as post-surgical analgesia, and haloperidol (20.6%). A key complication noted was pressure sores in 12 (9.1%) of the restrained patients. Nurse to patient ratio on wards where restraint was used ranged from 1:2 to 1:10.

### Nurses

Of the one hundred fifty nurses who completed the questionnaire, 133 (89%) were female. Mean age (SD) was 38.8 years (9.2), (range 18–59). The majority of the nurses (58%) had been working in the hospital for 5–6 years. The proportions of nurses reporting a specific method of restraint generally used in the wards were: bed rails 95%, straps 53%, bedding 43%, boxing gloves 18% and restraining belts 12%. Only 13% of the nurses reported having received training as students on the use of physical restraint; 3% reported receiving such training in service. In both cases the training amounted to less than 8 hours in total. Only 39% of nurses knew of a hospital policy on the use of restraint which they could consult if needed. When asked where the policy is kept, 17% reported that it was kept in the nursing manager’s office, 20% stated at the nursing station and 22% stated in the superintendent’s office.

### Medical practitioners

Fifty-nine doctors completed the questionnaire; 35(59%) were male. Mean age (SD) was 31 years (4.6) (range 23–43). Half had worked in the study hospital for ≥ 3 years. A majority (83%) had specialised in internal medicine. A majority (78%) had ordered restraint for a patient at some time, but only 36% reported having had guidance on the use of restraint. More than 90% were unaware of the existence of a hospital policy on restraint.

**FIGURE 1:**
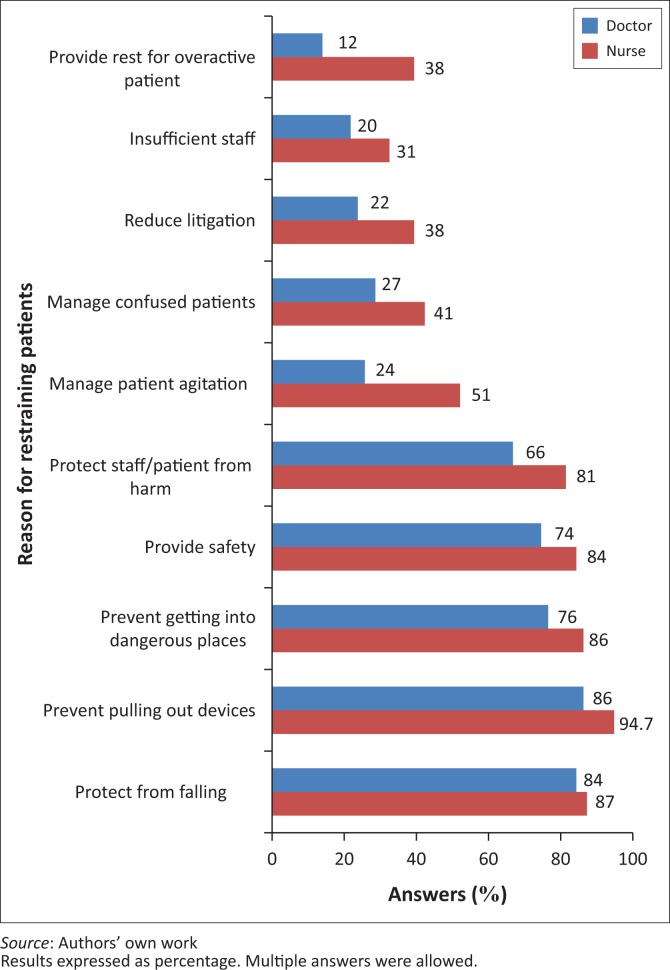
Reason for restraining patients as given by nurses and doctors.

Key reasons given by both nurses and doctors for the use of restraint in patients were to prevent patients from falling, to prevent them from removing devices, to prevent them from going to dangerous places, to ensure patients’ safety and to protect both patients and staff (Figure 1). When asked whose decision it was for a patient to be restrained, 53 (90%) of doctors and 143 (95%) of nurses stated it was the doctor’s decision; 36 (71%) of doctors and 64 (43%) of nurses stated it was that of a professional nurse; and a minority stated it was that of an enrolled or auxiliary nurse, or a rehabilitation therapist. A majority of nurses and doctors were of the opinion that such a decision was a joint responsibility of the nurse and the doctor.

**FIGURE 2:**
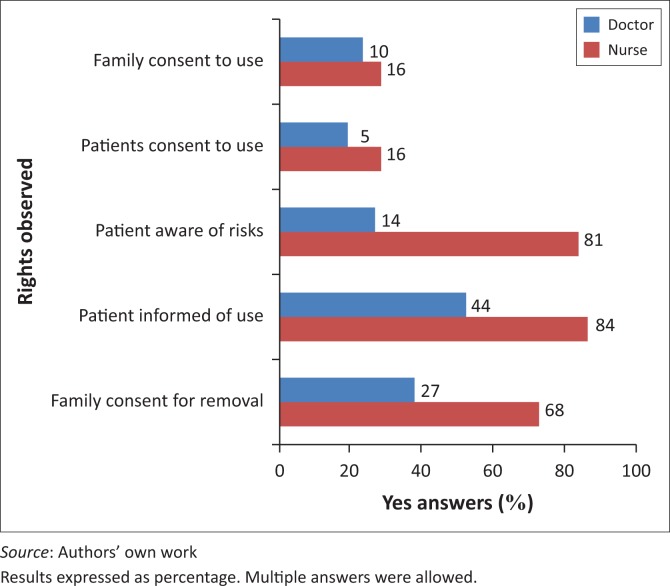
Protection of patients’ rights in use of restraint, as reported by doctors and nurses (percentages ‘yes’ responses).

**FIGURE 3:**
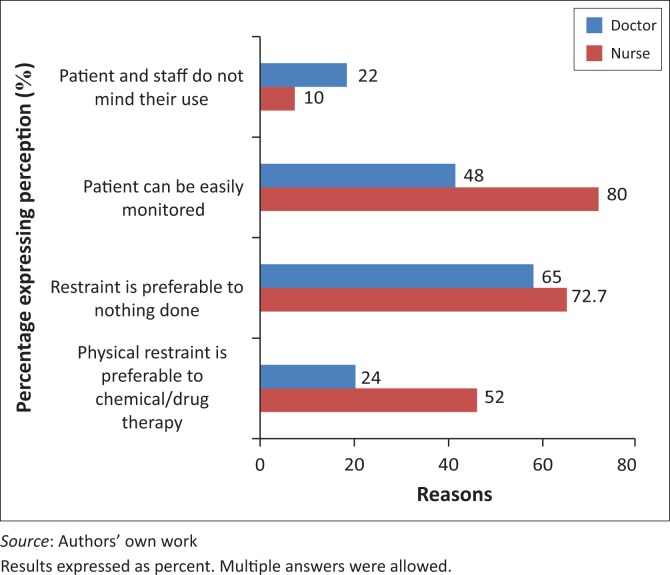
Reasons for doctors’ and nurses’ preference for use of physical restraint or chemical restraint.

More doctors (98%) than nurses (74.7%) contended it was the responsibility of a RN to review restrained patients. When asked whether a family member or a legal representative has a say in the use of restraint, and whether patients are informed of a need for them to be restrained, more nurses (16%) than doctors (10%) reported that such representatives and patients were informed of risks involved. However, a majority of nurses and doctors reported that no consent was obtained from patients or a family or legal representative before restraint was used (Figure 2). More nurses (76%) than doctors (48%) reported a preference for the use of physical restraint, whereas 52% of doctors preferred the use of chemical restraint. A reason commonly given by nurses for a preference for the use of physical restraint was that it does not cause dependency in the patient and that it is easier to monitor these patients (Figure 3). When asked to what extent they approved of the use of physical restraint to protect patients from self-harm, a majority of doctors and of nurses responded favourably, but only 37% of doctors and 52% of nurses approved of the use if it is to protect the hospital from possible litigation.

## Discussion

The study found a prevalence of 23% use of physical restraint. Slightly over half of the restrained patients were in acute general medical wards. An interesting finding was the absence of restrained patients in psychiatry wards. Nurses and doctors in psychiatry reported that they do not restrain patients to the bed. Patients that cannot be contained are placed in an isolation room until they calm down. The prevalence of use of physical restraint in acute hospitals reported in other studies ranges between 3% and 25% (Agens 2010; Evans *et al*. 2002; Krüger 2013). Krüger *et al*. (2013) found variation in the prevalence across wards with an overall prevalence of 12%. Evans *et al*. 2002 reported a prevalence of 7.4% to 17% use in acute care hospitals and up to 37% in long-term care in the United States. Comparison of prevalence rates of restraint use in acute care is challenging because of different definitions of physical restraint, and variation in data collection methods, settings and populations investigated. Some studies, for example, include intensive care units, whereas others do not (Krüger *et al*. 2013).

In the present study, the use of physical restraint was greater in medical wards where acute medical conditions are precipitants of delirium, and resultant confusion and agitated behaviour. The use of chemical restraint in the present study was higher in surgical wards, where it has an advantage of simultaneously providing post-surgical analgesia. Some medical conditions like sepsis may cause confusion in patients. A diagnosis of confusion was a risk factor that predicted the use of both physical and chemical restraint on patients.

Half of the patients in the study were restrained for other reasons, such as to prevent a fall or interference with treatment. Congruent with findings of researchers in high income countries (Evans & FitzGerald 2002; Karlsson *et al*. 2001), our study, too, found that health professionals were inclined to use physical restraint to protect confused patients from self-harm, such as falling, and removing intravenous and/or feeding tubes (Demir 2007; Heinze *et al*. 2012; Martin & Mathisen 2005; Minnick *et al*. 2007). Interestingly, no patient records in the present study reported a fall incident in a restrained patient; hospital policy requires any such incident to be recorded and reported. This finding is inconsistent with findings recorded by other researchers (Capezuti *et al*. 1996; Watson 2001), where restrained patients had been found to fall out of bed while struggling to free themselves from restraints.

The use of restraint may have both physical and psychological sequelae (Irish Nurses Organisation 2003). A complication commonly identified in our study, and reported by the participants to be associated with restraint, was pressure sores and abrasions, which were recorded in 12% of the patients. Guidelines on steps to be followed in the use of restraints in high income countries include an evaluation of the type of restraint to be used and its potential to cause injury; and the least restrictive to be used for the shortest duration necessary. The need for restraint and the type of restraint to be used should be a decision of a multidisciplinary team including, where possible, doctors, nurses, an occupational therapist and a physiotherapist (Irish Nurses Organisation 2003). The choice of restraint and its use should be documented in the medical record, and the application of restraints should be done or supervised by a RN; the latter guideline was neither clearly indicated nor followed in the present study.

A restrained patient is unable to provide for his or her basic needs, which include turning, eating, drinking and toileting (Maccioli *et al*. 2003). Health professionals who care for restrained patients must therefore be skilled in providing for these needs, while simultaneously monitoring the patient for complications arising from restraint.

Bed rails, belts and chairs with an attached table are reported in the literature as the most commonly used types of restraint (Minnick *et al*. 2007). The most commonly used types of restraint in our study were bed rails, straps (belts) and bedding. Boxing gloves (mittens) may be a preferable mode of restraint as they are less restrictive for a patient (Maccioli *et al*. 2003), yet are effective in preventing a patient from disrupting a therapeutic device. The commonest mode of restraint in this study was double bed rails (93%). A single side bed rail if used to assist the patient to move is not regarded as a restraint. Double rails or a single rail but with an open side against the wall are regarded as restraints as they restrict movement. Bed rails have been regarded as a cause of trauma as patients climb over them and fall from a greater height compared to falling from bed (Evans *et al*. 2003). Analgesics, sedatives and neuroleptics used for the treatment of pain, anxiety or psychiatric disturbance may be used as agents to mitigate a need for restraining therapy for a patient; such options were underused in the present study, with a clear preference shown for physical restraint.

American guidelines on the use of physical restraint (Maccioli *et al*. 2003) state that the initial doctor’s order may be a verbal order based on an assessment of the patient by a RN that has been communicated to the doctor. Strictly, the verbal order should be followed as soon as possible with a bedside assessment of the patient by the doctor. The timing of the review varies depending on the state of the patient. When restraints are initiated for marked agitation or violence, the doctor should be notified of the use of restraint within an hour and the doctor should examine the patient within 4 hours. In cases where patients are restrained only to prevent interference with treatment, the doctor should be notified within 12 hours and the patient should be examined by the doctor within 24 hours. A decision to use restraint and its use were not clearly recorded in patient notes in our study. Neither was it indicated who had ordered their use.

American guidelines state that: ‘In general, a calm patient receiving restraining therapies must be monitored for complications at least every four hours. Agitated patients need more frequent monitoring, and re-evaluation every 15 minutes is recommended until the patient becomes calm’ (Maccioli *et al*. 2003). Complications of restraint include falls and injuries, death from strangulation, incontinence of urine and stool, decreased functional status, as well as psychological effects that include increased agitation, anxiety and depression (Cotter 2005). Although a pressure care chart was among documents in patient files in our study, specific patient monitoring and evaluation instructions were lacking.

The use of physical restraint and/or chemical restraint is reported to be associated with patient to nurse ratio (Irish Nurses Organisation 2003; Möhler & Meyer 2014). Research in certain countries has indeed shown that staffing patterns are a factor in the use of restraint on patients (Goethals *et al*. 2012; Happ 2000). In our study, no association was found between the nurse to patient ratio and the use of physical restraint; the use of restraint was found in acute general wards where the nurse to patient ratio was as high as 1:2 to those where the ratio was 1:10.

It has been previously reported that a decision of health professionals, nurses in particular, to use or not to use physical restraint on patients is frequently accompanied by feelings of ambiguity, frustration, powerlessness and unease (Goethals *et al*. 2012; Janelli, Stamps & Delles 2006). The availability of clear and standardised guidelines in the form of a policy document for these workers is therefore crucial in supporting them in such ethical and legal dilemmas (Köpke *et al*. 2012; RNs’ Association of Ontario (RNAO), 2012). Policy guidelines on the use of restraint in our study were inadequate and the majority of the health care workers were unaware of such guidelines.

No uniformly defined protocol for the management of restrained patients was available to these workers. A single page document, in the form of a written notice, was provided to guide nurses in the management of confused patients. This document, in line with international guidelines (Irish Nurses Organisation 2003; Maccioli *et al*. 2003), states that sedation and analgesia should be used in confused patients before the application of physical restraint. Thus, physical restraint should be applied only where chemical restraint is not effective or is contraindicated. The document also states that if a RN finds it necessary to use restraints, a doctor must be informed of this observation and the doctor must prescribe the restraining order in the patient’s notes. Moreover, the use of restraint must be explained to visiting relatives and the patient must be observed frequently. The document does not define the frequency of such observations, in order to create a uniform standard of nursing such patients; rather, it is prone to individual interpretation, as was found in the study.

## Limitations

Limitations of the study included the following. The study was cross-sectional, and the prevalence of the use of restraint and resultant complications was not recorded prospectively. Although complications such as pressure sores were recorded, temporal relationship to restraining cannot be conclusive. Data were collected during the day and during a single eight-hour shift (08:00–16:00); wards were not visited at night and a comparison could not be drawn on the use of restraint during day and night shifts. The majority of patients were too ill to be interviewed on their experience of being restrained. Family members were not interviewed on their views of the use of restraint on their relative, which would have provided a family perspective in future patient management. The findings are not generalisable as the study was limited to one hospital but the study would stimulate others to review their procedures in restraint use.

## Recommendations

All involved in the care of patients should strive to keep the use of restraints to a minimum.

There is a need for ongoing training of health care workers on the indications, risks, complications and procedures to be followed in the use of restraints in order to empower health care workers in making informed decisions where such type of therapy is unavoidable.

The prescription and application of restraints on patients should be done by specially authorised health care practitioners.

Family, carers and where possible the patient should be involved and informed of the need and management of restraint therapy.

The monitoring of patients on whom restraint therapy is applied needs to be individualised to the patient’s condition and needs.

Clear hospital policy and comprehensive guidelines should be available to guide and support health care workers in situations where the use of restraint therapy is unavoidable.

## Conclusions

Restraint-free management of confused and other vulnerable patients would be an ideal situation to aspire to, but the use of restraints is sometimes unavoidable. The descriptive study showed a high proportion of physical restraint use in patients in the acute hospital wards but no clear policy to guide their use. Restraints were used largely to prevent harm and to protect devices. There was little education on the use of this mode of patient management and a lack of patients’ and family or caregivers’ involvement in decision for their use. There was a lack of a coordinated approach in the management of such patients. Health care personnel, including doctors, rehabilitation therapists and nurses, require continuing education on alternative measures to take to manage vulnerable patients. A policy on the use of restraint and comprehensive guidelines for its use should be developed and made available to guide health care practitioners in the management of patients where restraint cannot be avoided.
